# A Prospective Pilot Study for Prognosis of Cardiac Resynchronization Therapy Super-Response Using Electrical and Mechanical Dyssynchrony Assessment in Patients with Heart Failure and Strauss Left Bundle Branch Block Criteria

**DOI:** 10.3390/life15040605

**Published:** 2025-04-05

**Authors:** Tariel Atabekov, Andrey Smorgon, Anna Mishkina, Sergey Krivolapov, Svetlana Sazonova, Mikhail Khlynin, Roman Batalov, Sergey Popov

**Affiliations:** Cardiology Research Institute, Tomsk National Research Medical Center, Russian Academy of Sciences, Kievskaya St., 111a, Tomsk 634012, Russia; sav@cardio-tomsk.ru (A.S.); anna123.2013@gmail.com (A.M.); cardiorhythm@mail.ru (S.K.); sazonova_si@mail.ru (S.S.); mskhlynin@mail.ru (M.K.); romancer@cardio-tomsk.ru (R.B.); psv@cardio-tomsk.ru (S.P.)

**Keywords:** heart failure, left bundle branch block, electrical dyssynchrony, mechanical dyssynchrony, electrocardiography, speckle-tracking echocardiography, cardiac scintigraphy, cardiac resynchronization therapy, super-response

## Abstract

Electrical and mechanical dyssynchrony (MD) underlies left ventricular (LV) contractile dysfunction in patients with heart failure (HF) and left bundle branch block (LBBB). In some cases, cardiac resynchronization therapy (CRT) almost completely reverses LV contractile dysfunction. The LBBB electrocardiographic Strauss criteria and MD assessment were proposed to improve CRT response. However, using these techniques separately does not improve LV contraction in 20–40% of patients after CRT device implantation. We aimed to evaluate whether the combined use of electrocardiography (ECG), speckle-tracking echocardiography (STE) and cardiac scintigraphy could improve the prognosis of CRT super-response in patients with HF and Strauss LBBB criteria during a 6-month follow-up period. The study prospectively included patients with HF, classified as New York Heart Association (NYHA) functional class (FC) II–III in sinus rhythm with Strauss LBBB criteria and reduced left ventricular ejection fraction (LVEF). Before and 6 months after CRT device implantation, ECG, STE and cardiac scintigraphy were performed. The study’s primary endpoint was the NYHA class improvement ≥ 1 and left ventricle end systolic volume decrease > 30% or LVEF improvement > 15% after 6 months of CRT. Based on collected data, we developed a prognostic model regarding the CRT super-response. Out of 54 (100.0%) patients, 39 (72.2%) had a CRT super-response. Patients with CRT super-response were likelier to have a greater S wave amplitude in V_2_ lead (*p* = 0.004), higher rates of global longitudinal strain (GLS) (*p* = 0.001) and interventricular delay (IVD) (*p* = 0.005). Only three indicators (S wave amplitude in V_2_ lead, GLS and IVD) were independently associated with CRT super-response in univariable and multivariable logistic regression. We created a prognostic model based on the logistic equation and calculated a cut-off value (>0.73). The resulting ROC curve revealed a discriminative ability with an AUC of 0.957 (sensitivity 87.2%; specificity 100.0%). The electrical and mechanical dyssynchrony assessment using ECG, STE and cardiac scintigraphy is useful in the prediction of CRT super-response in patients with HF and Strauss LBBB criteria during a 6-month follow-up period. Our prognostic model can identify patients who are super-responders to CRT.

## 1. Introduction

The abnormal electrical activation in patients with heart failure (HF) and left bundle branch block (LBBB) may cause dyssynchronous left ventricular (LV) contraction. Electrocardiography (ECG) abnormalities, such as negative terminal deflection in leads V_1_ and V_2_ (QS or rS), a minimum QRS duration of 140 ms in men and 130 ms in women and the presence of mid-QRS notching, are used as indicators of electrical dyssynchrony in Strauss LBBB patients [1]. Besides electrical changes, mechanical function is often impaired in LBBB [2]. In LBBB, the electrical activation reaches first the right ventricle, and then the electrical front usually transmits through the interventricular septum towards the LV lateral wall [2,3]. This abnormality in electrical conduction can result in mechanical dyssynchrony (MD), myocardial remodeling and decreased cardiac function and HF [2]. Moreover, MD does not equal electrical dyssynchrony and can also occur in its absence [4]. However, correlation between electrical and mechanical dyssynchrony has been previously reported, and current guidelines for cardiac resynchronization therapy (CRT) suggest indications that are based primarily on electrocardiographic criteria, such as QRS duration and QRS morphology [5]. The LBBB electrocardiographic Strauss criteria were proposed to improve CRT response. Nevertheless, the rate of nonresponse to CRT among patients with HF who meet the Strauss ECG criteria remains as high as 20% to 40% [6,7].

Nevertheless, MD assessment is important because it is an early marker of myocardial damage, a clinical risk factor of poor outcome and a predictor of CRT response. Additionally, it can be measured using a variety of imaging modalities. As shown in previous studies, speckle-tracking echocardiography (STE) has emerged as a promising and widely used method for MD assessing [8,9,10,11]. But, results of the PROSPECT (Predictors of Response to CRT) and Echo-CRT (Echocardiography Guided Cardiac Resynchronization Therapy) trials make echocardiography a controversial tool in MD assessment when selecting patients for CRT [12,13].

Several studies have reported on the prognostic significance of MD evaluated through cardiac scintigraphy in candidates for CRT with HF [14,15]. In these studies, only the global dyssynchrony of left ventricle was evaluated, without considering the features of regional contractility. However, many of the factors they concentrated on are isolated and offer limited advantages for clinical practice.

Electrical and mechanical dyssynchrony assessment using electrocardiographic, echocardiographic and radionuclide methods has been suggested for adding value to the selection of CRT candidates [2]. There are no studies on the combined use of electrical and mechanical dyssynchrony indicators based on the results of ECG, STE and cardiac scintigraphy. Currently, a limited number of studies have established a predictive model that can distinguish patients with a super-response to CRT [16,17,18]. However, using these techniques separately does not increase the number of patients with LV contraction improvement after CRT device implantation. We aimed to evaluate whether the combined use of electrocardiographic, echocardiographic and radionuclide methods could improve the prognosis of CRT super-response in patients with HF and Strauss LBBB criteria during a short-term follow-up period.

## 2. Materials and Methods

### 2.1. Study Design and Participants

**Figure 1:**
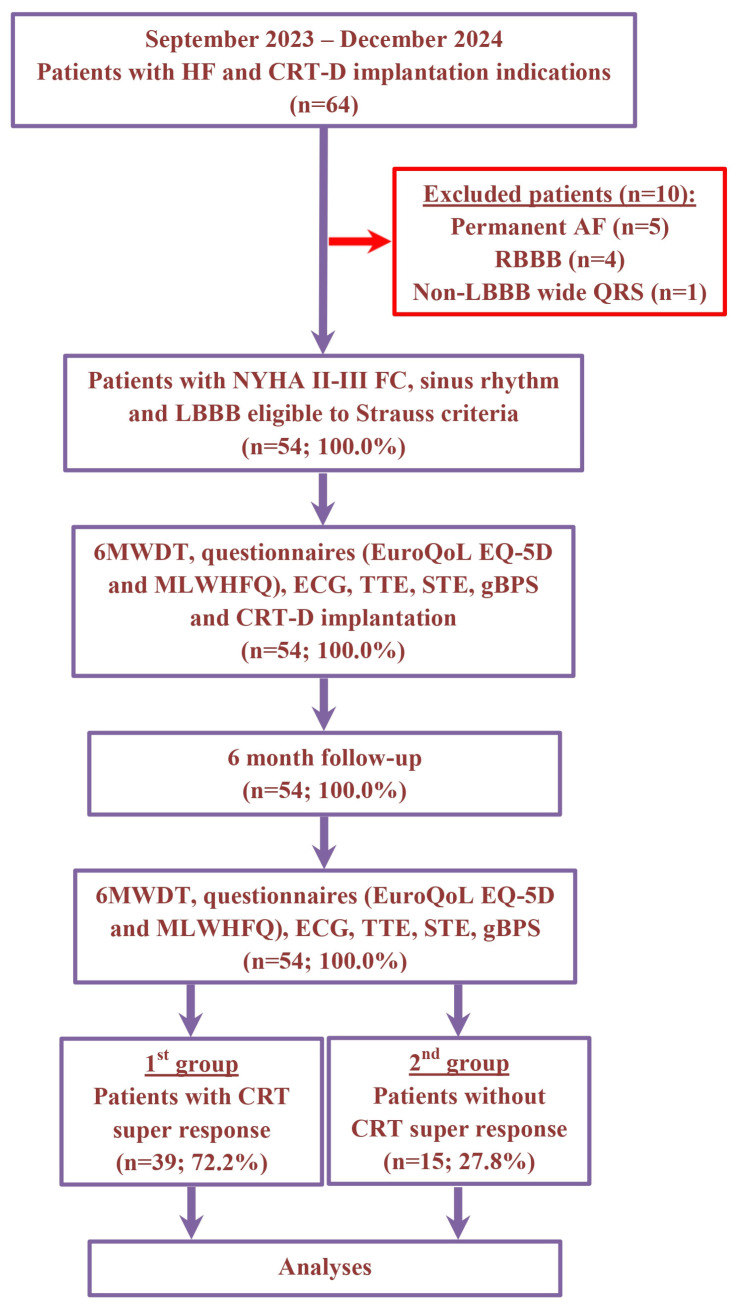
Schematic summary of enrollment flow and design of study. Abbreviations: 6MWDT, 6 min walk distance test; AF, atrial fibrillation; CRT, cardiac resynchronization therapy; CRT-D, cardiac resynchronization therapy devices with the defibrillation function; ECG, electrocardiography; EuroQoL EQ-5D, European Quality Of Life Group Questionnaire; FC, functional class; gBPS, gated blood pool SPECT; HF, heart failure; LBBB, left bundle branch block; MLWHFQ, Minnesota Living With Heart Failure Questionnaire; NYHA, New York Heart Association; RBBB, right bundle branch block; STE, speckle-tracking echocardiography; TTE, transthoracic echocardiography.

The non-randomized, prospective, clinical study recruitment occurred between September 2023 and December 2024 at the Department of Surgical Arrhythmology and Cardiac Pacing of the Cardiology Research Institute. The study included a total of 64 patients with HF, classified as New York Heart Association (NYHA) functional class (FC) II–III, with a left ventricular ejection fraction (LVEF) of 35% or less and a wide QRS duration (≥130 ms for women and ≥140 ms for men) with LBBB. All participants met the Strauss criteria and had indications for the implantation of CRT devices with defibrillation function (CRT-D). The etiology of HF was classified as ischemic if there was significant coronary artery disease present (defined as ≥50% stenosis in one or more of the major coronary arteries based on coronary angiography or computed tomography angiography) and/or a history of myocardial infarction or previous revascularization procedures. Patients were excluded if they had persistent atrial fibrillation, right bundle branch block, wide QRS with a non-LBBB pattern, hypertrophic cardiomyopathy, HF decompensation, a recent myocardial infarction (within the last 3 months), acute myocarditis, a previously implanted cardioverter-defibrillator or pacemaker, severe comorbidities, cognitive impairments, required revascularization or heart transplantation, were pregnant or were under 18 years of age (Figure 1).

Each patient filling out questionnaires (the European Quality Of Life Group Questionnaire [EuroQoL EQ-5D] and the Minnesota Living With Heart Failure Questionnaire [MLWHFQ]) and underwent a comprehensive physical examination, which included a 6 min walk distance test (6MWDT), ECG, STE, transthoracic echocardiography (TTE), Holter ECG monitoring, coronary angiography, blood tests and gated blood pool single-photon emission computed tomography (gBPS), both before and after the implantation of the CRT-D at the 6-month mark. In all instances, the CRT-D was implanted in accordance with the most recent guidelines [5]. All patients were provided with standard therapy in line with the current HF management guidelines [19]. The follow-up was conducted six months after the implantation of the CRT-D.

### 2.2. Acquisition and Analysis of Electrocardiograms

One experienced investigator assessed the standard supine 12-lead ECGs recorded at a speed of 25 mm/s and amplitude of 10 mm/mV taken before and after the implantation of the CRT-D. The following data were gathered: alpha angle; PQ interval; corrected QT interval [20]; corrected QT interval adjusted for LBBB or biventricular pacing (BP); QRS duration; QRS notching/slurring observed in leads I, aVL, V_5_, V_6_, QS or rS in V_1_ and V_2_; S wave amplitude in leads V_1_–V_3_ and V_6_; and q wave amplitude in leads I, aVL, V_5_ and V_6_.

### 2.3. Acquisition and Analysis of Echocardiograms

Single-blinded specialists, who were unaware of the CRT super-response status, conducted the analysis of TTE. The TTE, along with the assessment of intracardiac hemodynamic parameters, was conducted using a Philips HD15 PureWave ultrasound machine (Philips Ultrasound, Inc., Bothell, WA, USA) both at baseline and six months following the implantation of the CRT-D. The examination was conducted from standard positions, measuring the volumetric and indexed parameters of the heart chambers, as well as the LVEF and systolic pressure in the right ventricle (RV). The functions of the mitral, tricuspid and aortic valves, along with the contractility of the ventricles, were evaluated. TTE was conducted in accordance with the current guidelines [21]. The early diastolic filling velocity (E wave), peak filling velocity during atrial systole (A wave), E/A ratios and e′ values were calculated. The average of the lateral and septal e′ values was obtained, and E/e′ ratios were recorded.

The STE for assessing the intracardiac hemodynamics of the heart was performed using the Affinity 70 CV ultrasound system (Philips Ultrasound, Inc., Bothell, WA, USA). High-frequency sensors with a phased array (3–8 MHz) were used. The study was conducted in standard B-M-D modes. All native data were stored on a production server with subsequent processing by the Q-lab Ver15.7 system. The STE was performed in the Q-lab 15 post processing software package 7. To obtain optimal images, the study was performed at a frame rate of 60 to 110 frames per second, with breath retention (if possible) and an even ECG. In B-mode, video loops were recorded from apical access (four-chamber, two-chamber and three-chamber positions) and from parasternal access (the short LV axis at the MV level, at the papillary muscle level and at the apical segment level). The following indicators were evaluated in this research:

1. Global longitudinal strain (GLS)—longitudinal deformation is a deformation of the myocardium directed from the base to the tip of the heart. During systole, the ventricular fibers of the myocardium shorten with translational movement from the base to the tip.
2. Global circumferential strain (GCS)—circular deformation is a shortening of the LV myocardial fibers along the circular perimeter in the plane of the short axis of the heart.
3. RV free wall strain (RVFWS)—deformity of the RF and the free wall of the RV was measured in the apical four-chamber position using AutoStrain RV.

### 2.4. Acquisition of Cardiac Scintigraphy Data

The scintigraphy examination was conducted using CZT single-photon emission computed tomography/computed tomography (SPECT/CT) (GE Discovery 570C, GE Healthcare, Haifa, Israel), equipped with low-energy multi-pinhole collimators and 19 stationary detectors. Each detector was composed of 32 × 32 pixelated CZT elements, each measuring 2.46 × 2.46 mm. The energy window was symmetrically centered around ±20% of the 140 keV photopeak of ^99m^Tc. The images were reconstructed using a dedicated workstation (Xeleris 4.0; GE Healthcare, Haifa, Israel).

gBPS was conducted after the in vivo labeling of the patient’s red blood cells with a dose of 555–720 MBq of ^99m^Tc-pertechnetate [22]. Data were collected using ECG-gating with 16 frames per cardiac cycle. Patients were placed in a supine position with their arms raised above their heads for an acquisition duration of 10 min. No attenuation correction was applied. Images were reconstructed using iterative reconstruction (60 iterations; Green OSL α 0.7; Green OSL β 0.3) and a Butterworth post-processing filter (frequency 0.52; order 5) in a 70 × 70 pixel matrix with 57 slices. Image quantification and phase analysis were performed using Quantitative Blood Pool SPECT 2009.0 software (Cedars-Sinai Medical Center, Los Angeles, CA, USA), which enabled the assessment of functional variables for the left and right ventricles. Ventricular contours were manually adjusted as needed. The following parameters were determined for both ventricles: peak ejection rate (PER, expressed as EDV/s), peak filling rate (PFR, EDV/s) and second peak filling rate (PFR2, EDV/s).

The severity of intra- and interventricular dyssynchrony was assessed using Fourier transform. Global MD was evaluated through indices such as phase standard deviation (PSD), histogram bandwidth (HBW) and phase entropy (PE) for both ventricles. Interventricular dyssynchrony (IVD) was determined based on the histogram peak of the left and right ventricles. Additionally, intraventricular dyssynchrony (ID) for both ventricles and regional MD indices (SD and E) were analyzed. The regional analysis of left ventricular MD focused on the assessment of the free wall (FW) and the anterior (AW), lateral (LW), inferior (IW) and septal (SW) walls of the LV. The average effective radiation dose for the entire study protocol was 7.48 ± 1 mSv (ranging from 5.1 to 10.3 mSv) per patient.

### 2.5. Implantation and Programming of the CRT-D

The active fixation atrial and defibrillation leads were positioned at the appendage of the right atrium and the septum or apex of the RV, respectively. The passive fixation leads for the left ventricle were implanted in the lateral, posterolateral or anterolateral cardiac vein. The implantation of the leads was carried out at the discretion of the implanting physician, following a standard procedure under fluoroscopic guidance via a transvenous approach. The positions of the leads were verified through fluoroscopy in both postero-anterior and left anterior oblique views, as well as through intraoperative threshold testing. The capture threshold and sensing amplitude of the leads were measured using a pacing system analyzer (Medtronic, Minneapolis, MN, USA) with sterile crocodile clip cables.

CRT-D programming was performed in accordance with international guidelines [23]. In each CRT-D device, the monitoring zone was set to a heart rate of 140–170 beats per minute (bpm) for more than 50 consecutive cycles without the use of antitachycardia pacing (ATP) or shock therapy. The ventricular tachycardia (VT) zone was configured for 170–200 bpm with a duration of 30 cycles, incorporating ATP (with at least one burst pacing and one ramp pacing) and shock therapy (the first shock delivered at a submaximal discharge level). The ventricular fibrillation (VF) zone was programmed for 201–240 bpm with 12 cycles, including ATP during CRT-D charging and the maximum shock discharge.

### 2.6. Definition of the CRT Super-Response Criteria

The criteria for a super-response to CRT were established as a combination of clinical and echocardiographic improvements. Clinically, this included the absence of HF related hospitalizations and an improvement of at least one class in the NYHA classification. Echocardiographically, it required either an increase in LVEF of 15% or more or a reduction in LV end-systolic volume of 30% or more from baseline to the six-month follow-up [14,24].

### 2.7. Study Endpoint

The primary endpoint of the study was the presence of a super-response to CRT at 6 months after device implantation.

### 2.8. Statistical Analysis, Risk Assessment and Score Creation

Categorical and qualitative variables were expressed as counts (n) and percentages (%), while normally distributed continuous variables were reported as the mean (M) ± standard deviation (SD). Non-normally distributed variables were presented as the median (M_e_) along with interquartile ranges [Q_1_ and Q_3_]. The normality of continuous data distribution was assessed using the Kolmogorov–Smirnov, Lilliefors and Shapiro–Wilk tests. Differences between groups for continuous data were evaluated using the two-sided Student’s *t*-test for normally distributed data or the Mann–Whitney U-test for independent ordinal or non-normally distributed data. For dependent samples, the Wilcoxon test was applied. The distribution of categorical and qualitative variables was analyzed using either Fisher’s exact test or the chi-square test.

For the primary outcome, logistic regression with stepwise elimination was used to distinguish the possible predictors of CRT super-response. We first performed a univariable logistic regression analysis to test the association among our primary endpoint (dependent variable) and all clinical outcomes (independent variables). Characteristics significantly (*p* < 0.05) related with the outcome according to univariable logistic regression were first introduced as potential variables in a multivariable logistic regression analysis. The multicollinearity was excluded using Spearman’s analyses. The test for collinearity was performed to exclude possible confounders between included independent variables. Goodness-of-fit was performed by the Hosmer–Lemeshow test. Correlations with significance among the predictors and other parameters were assessed using *t*-test and Pearson’s test. Our regression analysis results were presented as odds ratios (ORs) with 95% confidence intervals (CIs).

Finally, variables that were independently associated with our endpoint were included in the risk score. The area under the curve (AUC) was computed to assess the discriminative power of the risk stratification model.

All statistical analyses were carried out with statistical software Medcalc 19.2.6 (MedCalc Software, Ostend, Belgium) and Statistica 10.0, StatSoft (Tulsa, OK, USA), and statistical significance was determined by a *p*-value < 0.05.

## 3. Results

### 3.1. Characteristics of the Study Population

In 64 patients with HF and CRT-D implantation indication, we excluded 10 patients with permanent atrial fibrillation (*n* = 5), RBBB (*n* = 4) and wide QRS with non-LBBB morphology (*n* = 1). For the remaining 54 (100.0%) patients, the average age was 59.9 ± 9.8 years and 36 (66.6%) individuals were male. Six months following CRT-D implantation, a super-response was observed in 39 (72.2%) patients (1st group). The remaining 15 (27.8%) patients did not have a CRT super-response (2nd group). The patients’ demographic and clinical characteristics are presented in Table 1.

Individuals with a CRT super-response did not differ significantly from patients without a super-response regarding baseline demographics and clinical and postoperative characteristics.

### 3.2. EuroQoL EQ-5D, MLWHFQ and 6MWDT Characteristics

The two groups did not show any significant differences in terms of the baseline EuroQoL EQ-5D, MLWHFQ and 6MWDT indicators (Table 1). After 6 months of CRT, both groups showed significant improvement in all three measures. In the 1st group, the EuroQoL EQ-5D score improved from 57.1 ± 9.8 to 74.8 ± 8.1 (*p* < 0.001), the MLWHFQ was 58.7 ± 17.3 vs. 27.2 ± 15.5 (*p* < 0.001) and the 6MWDT was 295.4 ± 68.9 m vs. 438.5 ± 53.6 m (*p* < 0.001). In the 2nd group, EuroQoL EQ-5D improved from 59.3 ± 8.4 to 68.6 ± 8.2 (*p* = 0.003), the MLWHFQ was 61.4 ± 11.7 vs. 41.7 ± 17.6 (*p* < 0.001) and the 6MWDT was 301.4 ± 70.3 m vs. 413.4 ± 81.2 m (*p* < 0.001). It should be noted that the EuroQoL EQ-5D and MLWHFQ scores at 6 months after CRT were significantly better in the 1st group than in the 2nd (*p* = 0.025 and *p* = 0.008, respectively).

### 3.3. ECG Characteristics

Patients with super-response to CRT were more likely to have QRS notching in the V_6_ lead on the baseline ECG (100.0% vs. 80.0%, *p* = 0.004). The baseline amplitude of S waves in leads V_1_ and V_2_ was significantly increased in patients with CRT super-response compared to those without (1.8 ± 0.8 mV vs. 1.1 ± 0.5 mV, *p* = 0.006, and 2.7 ± 1.1 mV vs. 1.6 ± 0.9 mV, *p* = 0.004, respectively). The two groups did not show significant differences in the other pre-CRT-D implantation ECG characteristics (Table 2). Six months after CRT-D implantation, both groups exhibited significant changes in the PQ interval; QRS duration; QT_c_; S wave in V_1_–V_3_ and V_6_ leads; and q wave in I, aVL, V_5_ and V_6_ leads (Table 2).

### 3.4. Echocardiography Characteristics

The STE revealed a significantly more pronounced dyssynchrony according to GLS assessment (−7.8 [−10.1; −6.0] % vs. −10.0 [−12.3; −9.8] %, *p* = 0.001) in patients with CRT super-response. In TTE with the intracardiac hemodynamic parameters assessment, left ventricular end-diastolic dimension (66.4 ± 6.3 mm vs. 70.5 ± 8.1 mm, *p* = 0.047), left ventricle ejection fraction (29 [25; 31] % vs. 32 [28; 35] %, *p* = 0.013), stroke volume (66.2 ± 16.8 mL vs. 80.1 ± 18.4 mL, *p* = 0.019), right ventricle systolic pressure (31 [25; 42] mmHg vs. 37 [30; 57] mmHg, *p* = 0.038) and E/e′ (10.82 [8.25; 15.41] vs. 12.27 [11.33; 21.61], *p* = 0.046) were significantly reduced in patients with CRT super-response compared to those without. Also, patients with CRT super-response were more likely to exhibit the asynchronous contraction of the interventricular septum according to TTE assessment (41.0% vs. 13.3%, *p* = 0.039). The two groups did not show any significant differences in terms of the remaining pre-CRT-D echocardiographic characteristics (Table 3).

After 6 months of CRT, both groups showed significant (*p* < 0.001) improvement in all three STE (GLS, GCS and RVFWS) measures (Table 3). It should be noted that GLS, LVEF and LVESV at 6 months after CRT were significantly more improved in the 1st group than in the 2nd group (−9.7 [−11.5; −7.9] % vs. −12.5 [−14.7; −11.0] %, *p* < 0.001; 38.0 [35.0; 45.0] % vs. 32.0 [29.0; 35.0] %, *p* < 0.001; 113.4 ± 52.0 mL vs. 160.4 ± 59.1 mL, *p* = 0.008, respectively). The changes in the other echocardiography indicators are detailed in Table 3.

### 3.5. Cardiac Scintigraphy Characteristics

The groups were similar in terms of the main gBPS indicators before and after CRT implantation, except for IVD (*p* = 0.005), PSD RVID (*p* = 0.04), HBW LV (*p* = 0.032) and PSD LVIW (*p* = 0.021). Six months after CRT-D implantation, a significant improvement was observed in the following indicators of the left ventricular MD in the super-responder group: RVID (*p* = 0.036), LVID (*p* < 0.001), IVD (*p* = 0.003) and HBW LV (*p* < 0.001), as well as PSD LVSW (*p* < 0.001) and LW (*p* = 0.021) (Table 4).

In patients without CRT super-response a significant improvement was noted in the indicators of HBW LV (*p* = 0.002) and PSD LVSW (*p* = 0.001) (Table 4). It is worth noting that after 6 months, the LVID indicator in patients with super-response significantly decreased compared to those without (80.0 [50.0; 123.0] ms vs. 113.8 [81.8; 123.0] ms, *p* = 0.030).

### 3.6. Risk Stratification Analysis

**Figure 2:**
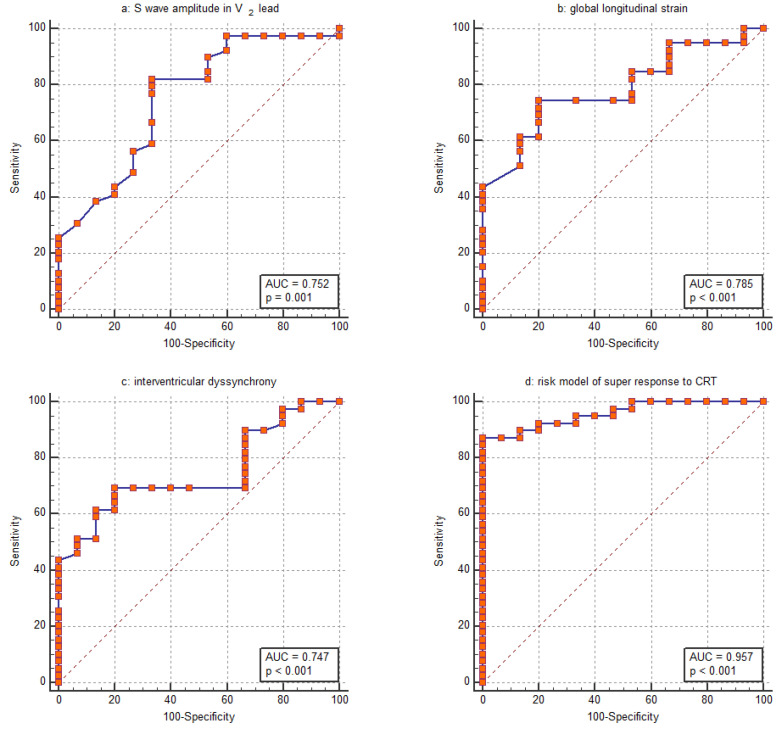
Receiver operating characteristic (ROC) curve for assessing the ability of (**a**) S wave amplitude in the V_2_ lead, (**b**) global longitudinal strain, (**c**) interventricular dyssynchrony and (**d**) the entire risk model to distinguish between patients with a super-response to CRT and those without.

Based on the assessed parameters, the GLS was the parameter most closely associated with the CRT super-response. Its discriminative ability was evaluated using ROC analysis revealing an AUC of 0.785 (95% CI: 0.652–0.885) (Figure 2b). The ability of the amplitude of the S wave in the V_2_ lead to distinguish the CRT super-response was comparable, with an AUC of 0.752 (95% CI: 0.616–0.860) (Figure 2a). Furthermore, this parameter strongly correlated with the amplitude of the S wave in the V_1_ lead (R = 0.820, *p* < 0.001). Regarding the IVD, the ROC curve analysis revealed an AUC of 0.747 (95% CI: 0.610–0.855) (Figure 2c). For GLS, logistic regression yields a negative coefficient, indicating that a higher GLS value decreases the likelihood of a CRT super-response. The corresponding ROC curve was inverted to allow for easier comparison with the other parameters (Figure 2b).

**Figure 3:**
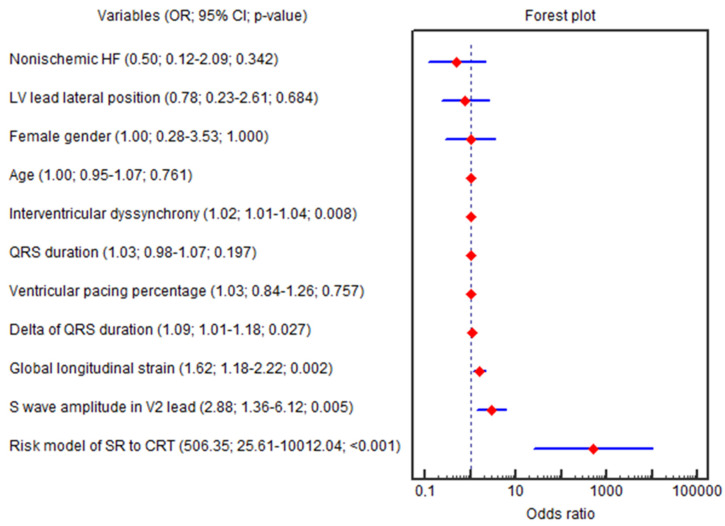
Forest plot for the univariable logistic regression results. Abbreviations: 95% CI, 95% confidence interval; CRT, cardiac resynchronization therapy; HF, heart failure; LV, left ventricle; OR, odds ratio; SR, super-response.

The GLS (OR = 1.62; 95% CI 1.18–2.22; *p* = 0.002), ∆QRS (OR = 1.09; 95% CI 1.01–1.18; *p* = 0.027), amplitude of the S wave in the V_2_ lead (OR = 2.88; 95% CI 1.35–6.12; *p* = 0.005), IVD (OR = 1.02; 95% CI 1.00–1.03; *p* = 0.008), baseline LVEF (OR = 0.81; 95% CI 0.69–0.96; *p* = 0.015), SV (OR = 0.95; 95% CI 0.92–0.99; *p* = 0.018), E/e′ (OR = 0.90; 95% CI 0.81–0.99; *p* = 0.045) and HBW LV (OR = 0.99; 95% CI 0.98–0.99; *p* = 0.037) were independently associated with the CRT super-response in univariable logistic regression (Figure 3).

Only three parameters (GLS, amplitude of the S wave in the V_2_ lead and IVD) remained significant in multivariable regression (OR = 2.35; 95% CI 1.35–4.10; *p* = 0.002; OR = 8.53; 95% CI 1.92–37.77; *p* = 0.004; OR = 1.03; 95% CI 1.00–1.07; *p* = 0.011, respectively), even after adjustment for age, female sex, QRS duration, nonischemic HF, LV lead lateral position and percentage of biventricular pacing at the 6th month (OR = 2.35; 95% CI 1.35–4.10; *p* = 0.002; OR = 8.53; 95% CI 1.92–37.77; *p* = 0.004; OR = 1.03; 95% CI 1.00–1.07; *p* = 0.011, respectively). The beta coefficients in the logistic equation correspond to the natural logarithm of the odds ratio (ln(OR) = β; OR = e^β^).

### 3.7. Development of a Risk Model

A risk assessment for the super-response to CRT was created using logistic regression based on the collected data. The GLS, amplitude of the S wave in the V_2_ lead and IVD were included in the final model since these parameters remained significant in multivariable logistic regression even after adjustment for age, female sex, QRS duration, nonischemic HF, lateral position of the LV lead and percentage of biventricular pacing at 6 months post-CRT. The AUC was computed to assess the discriminative power of the risk stratification score. The model showed strong discriminatory ability, indicated by an AUC of 0.957 (Figure 2d). With a cutoff value of greater than 0.73, the model demonstrated a sensitivity of 87.2% and a specificity of 100.0% in identifying patients with a super-response to CRT. Our risk stratification analysis indicated a positive predictive value of 92.3% and a negative predictive value of 73.3%. The percentage of cases correctly classified by the risk model was 87.0%.

**Figure 4:**
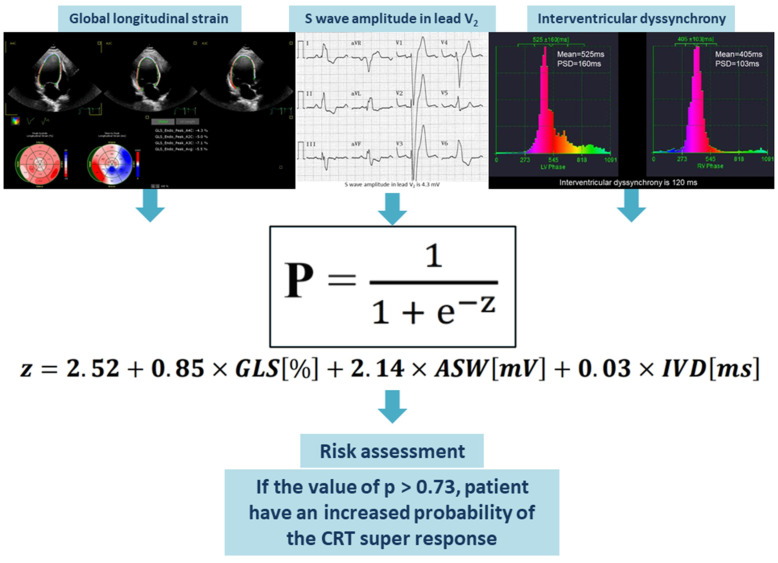
Visual representation of the suggested predictive model. Abbreviations: ASW, amplitude of S wave in the V_2_ lead; CRT, cardiac resynchronization therapy; GLS, global longitudinal strain; IVD, interventricular dyssynchrony.

The values of GLS (in %), amplitude of the S wave in the V_2_ lead (in mV), and IVD (in ms) should be entered into the assessment equation. The outcome of the logistic equation presented below is the probability (*p*) of a super-response to CRT. When the value of *p* exceeds 0.73, this risk assessment enables the identification of HF patients who have a higher likelihood of achieving a super-response to CRT (Figure 4).

Equation (1): Probability (*p*) of the CRT super-response. Abbreviations: ASW, amplitude of the S wave in the V_2_ lead; GLS, global longitudinal strain; IVD, interventricular dyssynchrony.

(1) $$p=\frac{1}{1+e^{-z}}\;\;\;\;\;\;\;\;\;\;\;\;\;\;\;\;\;\;\;\;\;z=2.52+0.85\times GLS\left[\%\right]+2.14\times ASW\left[mV\right]+0.03\times IVD\left[ms\right]$$

## 4. Discussion

In this prospective pilot study, we evaluated whether the combined use of electrocardiographic, echocardiographic and radionuclide methods could improve the prognosis of CRT super-response in patients with HF and Strauss LBBB criteria during a short-term follow-up period. We created a risk stratification model using standard clinical data (ECG and STE) and cardiac scintigraphy parameters to identify HF patients with an increased likelihood of a super-response to CRT. The suggested model is easy to apply and only needs a few elements, including ECG, STE and gBPS. This score should facilitate the identification of patients with a super-response, which may help in more accurately selecting candidates for CRT and lead to improved clinical outcomes.

Current CRT guidelines select patients mainly on electrocardiographic criteria such as QRS duration and morphology [5]. These criteria refer to the electrical dyssynchrony caused by a block of the left bundle branch as the substrate for CRT [25]. However, it has been shown that patients with LBBB morphology and wide QRS duration reveal variable ventricular activation patterns [25]. This heterogeneity in MD among LBBB patients is thought to be one of the reasons why a significant number of patients fail to respond to CRT [25]. Electrical and mechanical dyssynchrony underlie the contractile dysfunction of the LV and the subsequent development of HF in patients with LBBB. Consequently, parameters indicating these electrical and mechanical dyssynchrony processes were included in our analysis.

### 4.1. Association of Longitudinal Strain Assessed by STE with CRT Super-Response

There is a correlation between longitudinal strain and LV remodeling processes in CRT patients [26]. A meta-analysis of twelve studies involving a total of 1004 patients revealed that CRT responders had significantly better resting GLS values compared with non-responders (GLS mean difference −2.13 [−3.03; −1.23], *p* < 0.001) [26], almost similar to our results (−2.2 [−2.2; −3.8], *p* = 0.001). Kydd, A., et al. showed the incremental value of LV GLS to predict the occurrence of LV reverse remodeling after 6 months of CRT [27]. The univariate odds ratio of LV GLS to predict LV reverse remodeling was 0.8 (95% CI 0.79–0.97, *p* = 0.008) [27]. In addition, in 45 patients with non-ischemic cardiomyopathy treated with CRT, LV GLS was independently associated with LV reverse remodeling at follow-up (OR 4.1; 95% CI 3.1–5.5; *p* < 0.001) [28]. Data from the Multicenter Automatic Defibrillator Implantation Trial–Cardiac Resynchronization Therapy (MADIT-CRT) trial, involving 1077 patients with NYHA FC I-II HF symptoms, showed that the event rate (all-cause mortality or non-fatal HF events) per 100 patient-years increased significantly along with the impairment in LV GLS [29]. In multivariate analysis, patients with LV GLS ≤ −8.7% (more preserved) showed the greatest prognostic benefit (OR 0.43; 95% CI 0.28–0.67; *p* = 0.005) [29]. In another study, it was revealed that CRT responders showed higher absolute GLS values (*p* < 0.001) than non-responders [30]. Although CRT responders had higher absolute baseline GLS values, Kadoglou, N., et al. failed to find any independent association of absolute GLS (OR = 1.11; 95% CI 1.01–1.32; *p* = 0.660) at baseline with CRT response at 6 months regarding logistic regression analysis [30]. Significant differences in our study cohort were observed only for GLS among the three STE indicators. Our results showed that significantly more pronounced dyssynchrony was revealed according to GLS assessment in patients with CRT super-response compared to patients without super-response (−7.8 [−10.1; −6.0] % vs. −10.0 [−12.3; −9.8] %, *p* = 0.001). The univariable logistic regression showed that GLS (OR = 1.62; 95% CI 1.18–2.22; *p* = 0.002) was independently associated with the CRT super-response. The GLS remained significant in multivariable regression (OR = 2.35; 95% CI 1.35–4.10; *p* = 0.002), even after adjustment for age, female sex and other well-known super-response predictors. We used GLS in a risk stratification model because of its strong *p*-value. By incorporating this parameter into the risk score, the MD processes should be accurately reflected.

### 4.2. Predictive Value of ECG Parameters in CRT Super-Response Prognosis

Previous research indicated that the Strauss criteria were more effective in identifying patients who would benefit from CRT implantation [31,32]. However, another study found that out of the total number of CRT responders (n = 50), 52% (n = 26) did not meet the Strauss ECG criteria, while 48% (n = 24) did meet the Strauss ECG criteria, with a *p*-value of 0.463 [6]. Bertaglia, E., et al. demonstrated that a more stringent definition of LBBB did not enhance the response to CRT when compared to the existing definition provided by the American Heart Association [33].

Another parameter associated with the CRT super-response is the amplitude of the S wave in the V_2_ lead. The S wave in the V_2_ lead reflects the electrical activity of the heart, specifically the depolarization of the ventricles. It is part of the QRS complex in an ECG and indicates the downward deflection that occurs during ventricular depolarization. In the V_2_ lead, the S wave can provide information about the heart’s electrical conduction and can be useful in diagnosing various cardiac conditions. Our electrocardiographic examination revealed associations between CRT super-response and the amplitude of the S wave in leads V_1_ and V_2_, QRS notching in V_6_ and delta QRS, indicating the close correlation between electrical dyssynchrony and the LV remodeling processes. But, according to the results of further analysis, the parameters amplitude of the S wave in the V_1_ and V_2_ leads maintained a close correlation with the super-response to CRT. However, these two parameters equally reflect electrical dyssynchrony in patients with Strauss LBBB criteria [1], so these two parameters will be interdependent values. Since the predictors in risk stratification models should be independent of each other, we decided to use the amplitude of the S wave in the V_2_ lead due to its stronger *p*-value. There are no publications found regarding the relationship between the amplitude of the S wave in the right precordial leads and the super-response to CRT. According to the results of our study, an increase in the amplitude of the S wave in the V_2_ lead of more than 1.7 mV was a predictor of a super-response to CRT (AUC = 0.752; sensitivity = 82.05; specificity = 66.67; *p* = 0.001). The univariable logistic regression showed that amplitude of the S wave in the V_2_ lead (OR = 2.88; 95% CI 1.35–6.12; *p* = 0.005) was independently associated with the super-response to CRT. This parameter remained significant in the multivariable regression, even after adjustment for age, female sex and other well-known super-response predictors (OR = 8.53; 95% CI 1.92–37.77; *p* = 0.004). With the addition of this parameter to the risk model, the electrical dyssynchrony was taken into account.

### 4.3. Association of MD Assessed by gBPS with CRT Super-Response

The prognostic importance of mechanical dyssynchrony evaluated through cardiac scintigraphy has been highlighted in various studies [14,15,34]. gBPS combines can measure ventricular synchrony and can even predict CRT outcomes [35,36,37,38]. Badhwar, N., et al. recently used equilibrium radionuclide angiography for predicting outcomes in HF patients undergoing CRT, and they concluded that lower LV dyssynchrony and interventricular synchrony may predict positive response to CRT [39]. In our study, patients exhibiting a super-response were more likely to have elevated rates of IVD (*p* = 0.005), PSD RV intraventricular dyssynchrony (*p* = 0.04), PSD LV inferior wall (*p* = 0.021) and a HBW of LV (*p* = 0.032). According to the univariate and multivariate logistic regression, only IVD was independently associated with the CRT super-response, even after adjustment for age, female sex, QRS duration, nonischemic HF, LV lead lateral position and percentage of biventricular pacing at the 6th month (OR = 1.03; 95% CI 1.00–1.07; *p* = 0.011). According to our study, in patients with a CRT super-response, baseline IVD was more pronounced compared to patients without a CRT super-response (*p* = 0.005). In addition, baseline IVD was significantly higher than the IVD in patients with CRT super-response. This may indicate that the assessment of myocardial dyssynchrony may provide additional information for the most successful resynchronization therapy. But disagreements with previous studies underline the importance of these findings and the need for future large-scale studies.

### 4.4. Prognostic Model of CRT Super-Response

The development of a predictive model for super-responders to CRT has emerged as a critical advancement in improving patient outcomes in HF management. As a significant proportion of patients do not respond optimally to CRT, identifying those likely to benefit the most is essential for personalized treatment approaches. By focusing on specific clinical, electrocardiographic and echocardiographic parameters, such a model could enhance the selection process and optimize therapy efficacy. There is a notable scarcity of research dedicated to developing a scale or model for identifying super-responders to CRT. This limited focus hinders the advancement of personalized treatment strategies that could significantly enhance outcomes for HF patients. Goldenberg et al. identified characteristics linked to reverse remodeling after CRT [40]. The researchers developed a response score incorporating seven factors—female sex, nonischemic HF, LBBB, QRS duration of 150 ms or more, previous HF hospitalization, left ventricular end-diastolic volume of 125 mL/m^2^ or greater and left atrial volume of less than 40 mL/m^2^—to help identify patients who may benefit from CRT [40]. Their multivariate analysis revealed that for each 1-point increase in the response score (which ranges from 0 to 14), there was a 13% increase in the clinical benefit of CRT, with a statistically significant *p*-value of less than 0.001 [40]. Furthermore, a strong direct relationship was found between the risk reduction linked to CRT and the response score quartiles. Patients in the first quartile showed no significant risk reduction for HF or mortality with CRT (hazard ratio = 0.87; *p* = 0.52), while those in the second and third quartiles exhibited a 33% (*p* = 0.04) and 36% (*p* = 0.03) reduction in risk, respectively [40]. In contrast, patients in the highest quartile experienced a substantial 69% risk reduction (*p* < 0.001; *p* = 0.005), although it is important to note that this response score is complex and was only applicable to patients with an LVEF of 30% or less and milder HF symptoms. Maass et al. conducted a prospective study involving 240 patients who received CRT implantation [41]. They developed a predictive model called the CAVIAR score, which includes four variables: age, QRS area, interventricular mechanical delay and apical rocking. This score was effective in forecasting clinical outcomes, specifically HF hospitalizations and overall mortality. The findings suggest that the CAVIAR score can serve as a useful tool in predicting patient prognosis following CRT. Yanagisawa et al. conducted a retrospective study involving 80 patients who received CRT and identified three independent predictors of a super-response to CRT [16]. Their prognostic model combines the percentage of right ventricular pacing greater than 90% prior to CRT, the absence of a previous history of ventricular arrhythmias and a smaller left atrial diameter. This model enhances the ability to predict a super-response to CRT after six months of treatment. The findings suggest that these factors can be crucial in anticipating patient outcomes following CRT. Another study developed a predictive model that identified significant factors associated with an enhanced response to CRT [17]. The model considers left atrial size (LAE), QRS duration and LVEF with *p*-values, indicating strong associations, such as *p* < 0.01 for LAE and *p* < 0.05 for QRS duration. The QQ-LAE score showed a high predictive accuracy, with an AUC of 0.83, demonstrating its potential utility in clinical practice. These findings underscore the importance of these parameters in identifying patients who are likely to achieve a super-response to CRT. However, this model was found to be more suitable for a smaller, defined group of individuals. Its applicability is restricted to a specific subset of the human population. Based on the findings from our study, the multivariate logistic regression identified three independent predictors of the CRT super-response. These predictors included indicators of LV electrical dyssynchrony, specifically the amplitude of the S wave in the V_2_ lead, as well as MD indicators like GLS and IVD. These factors were incorporated into the final predictive model, which demonstrated strong discrimination capability with an AUC of 0.957. The model achieved an accuracy of 87.0% in accurately diagnosing CRT super-response within our study population. Patients who scored above the threshold value (>0.73) are likely to gain greater benefits from CRT-D implantation. It is important to acknowledge that no score or model can guarantee a 100% probability of an individual experiencing a super-response to CRT. We have developed this predictive model to help physicians assess the likelihood of a super-response before implanting CRT devices.

To address the risk of overfitting, we implemented several strategies during our analysis:

1. We carefully selected the variables included in the logistic regression model based on both clinical relevance and statistical significance. This selection process was guided by the existing literature and theoretical frameworks, ensuring that we focused on variables that are known to have a meaningful impact on the outcome of interest. By limiting the number of variables to those most pertinent, we aimed to reduce the complexity of the model and mitigate the risk of overfitting.
2. We employed a stepwise selection method, which allowed us to iteratively add or remove variables based on their contribution to the model’s predictive power. This approach helped us identify the most significant predictors while avoiding the inclusion of extraneous variables that could lead to overfitting.
3. We conducted a cross-validation procedure to assess the robustness of our model. By splitting the dataset into training and validation subsets, we were able to evaluate the model’s performance on unseen data. This process provided insights into how well the model generalizes beyond the sample used for fitting, thereby helping to identify any potential overfitting.
4. We reported the model’s performance metrics, including the area under the receiver operating characteristic curve, to provide a quantitative measure of its predictive accuracy. This metric allows for an assessment of the model’s ability to discriminate between outcomes, further supporting the validity of our findings.

In summary, we recognize the concern regarding overfitting in the context of our limited sample size and have taken several steps to mitigate this risk. By carefully selecting variables, employing stepwise selection, conducting cross-validation, and reporting performance metrics, we aimed to ensure the robustness and generalizability of our logistic regression model.

## 5. Conclusions

In conclusion, for patients meeting the Strauss criteria for left bundle branch block, HF and reduced LVEF, the integration of electrocardiographic, echocardiographic and radionuclide techniques can enhance the prediction of CRT super-response during a short-term follow-up period. The prognostic model based on indicators of left ventricular electrical and mechanical dyssynchrony can be used to predict super-response to CRT and identify the most appropriate patients for clinical treatment. The further validation of our predictive model should be conducted through independent prospective cohort studies.

## 6. Limitations

Our research has several limitations. One key limitation is that it was a nonrandomized, single-center investigation. Additionally, the short follow-up duration and the relatively small sample size may have decreased the robustness of the findings. It is also important to mention that patients were exposed to additional radiation due to the use of gSPECT.

We acknowledge that as a single-center pilot study with a modest sample size, the findings should indeed be interpreted with caution. This limitation is important to consider, as the results may not be generalizable to broader populations or different clinical settings. The modest sample size restricts the statistical power of our analyses, which may affect the reliability of the findings. Additionally, being a single-center study means that the results may be influenced by specific local practices, patient demographics and other contextual factors that may not be present in other settings. Therefore, while our findings provide preliminary insights, they should not be viewed as definitive conclusions.

To address this limitation, we suggest several avenues for external validation:

1. We recommend conducting multi-center studies that include a larger and more diverse patient population. This would help to assess the reproducibility of our findings across different healthcare settings and demographic groups.
2. Future research could focus on longitudinal studies that track outcomes over time, which would provide a more comprehensive understanding of the effects observed in our pilot study.
3. We encourage the use of randomized controlled trials to further validate our findings. Randomized controlled trials would allow for a more rigorous assessment of the interventions and their effects, minimizing biases that may arise in observational studies.

In summary, while our pilot study offers valuable preliminary data, we emphasize the need for further research to validate our findings in larger, multi-center and more diverse populations. This will enhance the robustness of the conclusions drawn and contribute to the overall body of evidence in this area.

## Figures and Tables

**Table 1:** Demographic and baseline clinical characteristics of patients in the overall population and categorized by group.

Demographic and Clinical Characteristics	Overall Population (n = 54)	1st Group Pts with CRT SR (n = 39)	2nd Group Pts Without CRT SR (n = 15)	*p*_2–3_
	1	2	3	
Age, year, M ± SD	59.9 ± 9.8	60.2 ± 10.1	59.3 ± 9.1	0.801
Male sex, n (%)	36 (66.6)	26 (66.6)	10 (66.6)	0.990
EuroQoL EQ-5D, score, M ± SD	57.7 ± 9.4	57.1 ± 9.8	59.3 ± 8.4	0.279
MLWHFQ, score, M ± SD	59.5 ± 15.8	58.7 ± 17.3	61.4 ± 11.7	0.824
6MWDT, m, M ± SD	299.7 ± 69.3	295.4 ± 68.9	301.4 ± 70.3	0.854
Heart failure etiology:				
Ischemic, n (%)	23 (42.6)	17 (43.6)	6 (40.0)	0.694
Non-ischemic, n (%)	31 (57.4)	22 (56.4)	9 (60.0)	0.347
New York Heart Association class:				
II, n (%)	26 (48.1)	18 (46.2)	8 (53.3)	0.647
III, n (%)	28 (51.9)	21 (53.8)	7 (46.7)	0.647
Arrhythmias prior to CRT-D implantation:				
Sustained VT, n (%)	3 (5.5)	3 (7.7)	0 (0.0)	0.284
Ventricular fibrillation, n (%)	2 (3.7)	1 (2.6)	1 (6.6)	0.497
Paroxysmal AF, n (%)	14 (25.9)	11 (28.2)	3 (20.0)	0.549
Comorbidities:				
Hypertension, n (%)	17 (31.5)	12 (30.7)	5 (33.3)	0.637
Diabetes mellitus, n (%)	11 (20.4)	8 (20.5)	3 (20.0)	0.977
BMI, kg/m^2^, M ± SD	28.5 ± 5.2	28.5 ± 5.0	28.5 ± 5.9	0.706
Dyslipidemia, n (%)	34 (62.9)	26 (66.6)	8 (53.3)	0.374
_e_GFR, ml/min/1.73 m^2^, M ± SD	72.4 ± 17.9	74.4 ± 18.2	67.3 ± 16.8	0.195
Stroke, n (%)	4 (7.4)	2 (5.1)	2 (13.3)	0.317
Therapy:				
Beta-blockers, n (%)	52 (96.3)	38 (97.4)	14 (93.3)	0.497
Loop diuretics, n (%)	26 (48.1)	19 (48.7)	7 (46.7)	0.902
MRA, n (%)	52 (96.3)	37 (94.9)	15 (100.0)	0.392
ARNi, n (%)	33 (61.1)	25 (64.1)	8 (53.3)	0.437
SGLT2i, n (%)	43 (79.6)	32 (82.1)	11 (73.3)	0.488
ACEI, n (%)	14 (25.9)	8 (20.5)	6 (40.0)	0.150
Antiplatelet agents, n (%)	29 (53.7)	19 (48.7)	10 (66.6)	0.244
Lipid-lowering treatment, n (%)	48 (88.9)	36 (92.3)	12 (80.0)	0.207
Angiotensin II receptor blocker, n (%)	6 (11.1)	3 (7.7)	3 (20.0)	0.207
Amiodarone, n (%)	21 (38.9)	15 (38.4)	6 (40.0)	0.927
Anticoagulants, n (%)	18 (33.3)	14 (35.9)	4 (26.6)	0.530
Ivabradine, n (%)	3 (5.5)	2 (5.1)	1 (6.6)	0.845
Left ventricular lead position:				
Lateral vein, n (%)	30 (55.5)	21 (53.8)	9 (60.0)	0.694
Posterolateral vein, n (%)	13 (24.1)	7 (17.9)	6 (40.0)	0.095
Anterolateral vein, n (%)	5 (9.2)	5 (12.8)	0 (0.0)	0.154
Posterior vein, n (%)	2 (3.7)	2 (5.1)	0 (0.0)	0.392

Data are expressed as n (%) for categorical and qualitative variables, and M ± SD or M_e_ [Q_1_; Q_3_] for continuous variables. Abbreviations: 6MWDT, 6 min walk distance test; ACEI, angiotensin-converting enzyme inhibitors; AF, atrial fibrillation; ARNi, angiotensin receptor neprilysin inhibitor; BMI, body mass index; CRT, cardiac resynchronization therapy; CRT-D, cardiac resynchronization therapy device with the defibrillator; _e_GFR, estimated glomerular filtration rate; EuroQoL EQ-5D, European Quality Of Life Group Questionnaire; MLWHFQ, Minnesota Living With Heart Failure Questionnaire; MRA, mineralocorticoid receptor antagonist; pts, patients; SGLT2i, sodium glucose co-transporter 2 inhibitors; SR, super-response; VT, ventricular tachycardia.

**Table 2:** Basic pre- and post-CRT-D implantation ECG indicators.

Characteristics	Pts with CRT SR (n = 39)			Pts Without CRT SR (n = 15)			*p*_1–4_	*p*_2–5_
	Baseline	6 m.	*p*	Baseline	6 m.	*p*		
	1	2	3	4	5	6		
PQ, ms	198.2 ± 38.4	135.3 ± 28.1	**<0.001**	193.6 ± 35.1	136.2 ± 32.1	**0.001**	0.931	0.735
QRS, ms	174.3 ± 14.5	141.7 ± 15.4	**<0.001**	168.0 ± 19.5	146.9 ± 16.7	**<0.001**	0.324	0.315
∆QRS, ms	-	18.6 ± 9.3	-	-	12.3 ± 7.3	-	-	**0.014**
QT_c_, ms	505.8 ± 28.1	478.2 ± 29.3	**<0.001**	495.1 ± 23.1	476.7 ± 26.8	**0.026**	0.199	0.884
cQT_c_, ms	433.0 ± 25.3	435.9 ± 23.3	0.485	428.5 ± 21.3	430.8 ± 24.0	0.711	0.629	0.685
AA, °	−30.0 [−40.0; −5.0]	−5.0 [−40.0; 125.0]	0.069	−38.0 [−48.0; −30.0]	−75.0 [−126.0; 90.0]	0.711	0.132	0.120
QRS notching/slurring in leads:								
I	38 (97.4)	-	-	14 (93.3)	-	-	0.497	-
aVL	38 (97.4)	-	-	15 (100.0)	-	-	0.562	-
V_5_	32 (82.0)	-	-	10 (66.6)	-	-	0.232	-
V_6_	39 (100.0)	-	-	12 (80.0)	-	-	**0.004**	-
QS in V_1_	15 (38.4)	-	-	8 (53.3)	-	-	0.332	-
rS in V_1_	24 (61.6)	-	-	7 (46.7)	-	-	0.332	-
QS in V_2_	7 (17.9)	-	-	3 (20.0)	-	-	0.874	-
rS in V_2_	32 (82.1)	-	-	12 (80.0)	-	-	0.874	-
S wave in leads:								
V_1_, mV	1.8 ± 0.8	0.8 ± 0.6	**<0.001**	1.1 ± 0.5	0.8 ± 0.6	**0.012**	**0.006**	0.877
V_2_, mV	2.7 ± 1.1	1.2 ± 0.8	**<0.001**	1.6 ± 0.9	1.3 ± 0.9	0.239	**0.004**	0.877
V_3_, mV	3.0 ± 0.9	1.8 ± 0.8	**<0.001**	2.5 ± 1.1	1.9 ± 1.0	0.083	0.170	0.976
V_6_, mV	0.0 [0.0; 0.2]	0.1 [0.0; 0.5]	**0.047**	0.1 [0.0; 0.4]	0.4 [0.0; 0.8]	**0.037**	0.185	0.102
q wave in leads:								
I, mV	0.0 [0.0; 0.0]	0.2 [0.0; 0.5]	**<0.001**	0.0 [0.0; 0.0]	0.1 [0.1; 0.5]	**0.005**	0.757	0.961
aVL, mV	0.0 [0.0; 0.0]	0.2 [0.1; 0.6]	**<0.001**	0.0 [0.0; 0.1]	0.2 [0.1; 0.5]	**0.002**	0.469	0.862
V_5_, mV	0.0 [0.0; 0.0]	0.0 [0.0; 0.2]	**0.003**	0.0 [0.0; 0.0]	0.0 [0.0; 0.2]	0.067	0.779	1.000
V_6_, mV	0.0 [0.0; 0.0]	0.1 [0.0; 0.5]	**<0.001**	0.0 [0.0; 0.0]	0.1 [0.0; 0.4]	**0.007**	0.984	0.892

Bold values indicate statistical significance. Values are expressed as mean ± SD and M_e_ [Q_1_; Q_3_] for continuous variables and n (%) for categorical variables. Abbreviations: AA, alpha angle; cQT_c_, corrected QT interval to account for left bundle branch block or biventricular pacing; CRT, cardiac resynchronization therapy; CRT-D, cardiac resynchronization therapy device with the defibrillator; ECG, electrocardiography; pts, patients; SR, super-response.

**Table 3:** Basic pre- and post-CRT implantation echocardiographic indicators.

Indicators	Pts with CRT SR (n = 39)			Pts Without CRT SR (n = 15)			*p*_1–4_	*p*_2–5_
	Baseline	6 m.	*p*	Baseline	6 m.	*p*		
	1	2	3	4	5	6		
GLS, %	−7.8 [−10.1; −6.0]	−9.7 [−11.5; −7.9]	**<0.001**	−10.0 [−12.3; −9.8]	−12.5 [−14.7; −11.0]	**<0.001**	**0.001**	**<0.001**
GCS, %	−8.0 [−8.9; −5.8]	−9.5 [−10.0; −8.0]	**<0.001**	−8.4 [−8.5; −7.2]	−10.5 [−11.0; −8.9]	**<0.001**	0.216	0.068
RVFWS, %	−12.2 [−14.0; −10.0]	−13.3 [−15.0; −11.0]	**<0.001**	−12.0 [−12.0; −11.0]	−13.3 [−13.3; −11.5]	**<0.001**	0.428	1.000
LVEDD, mm	66.0 ± 6.3	60.4 ± 8.7	**<0.001**	70.5 ± 8.1	68.5 ± 9.5	0.169	**0.047**	**0.009**
LVESD, mm	57.4 ± 6.1	49.3 ± 9.8	**<0.001**	61.1 ± 8.3	58.4 ± 8.7	0.046	0.182	**0.004**
LVEDV, mL	240.0 ± 60.4	184.5 ± 65.2	**<0.001**	258.1 ± 73.7	233.8 ± 78.8	**0.015**	0.353	**0.047**
LVESV, mL	173.7 ± 49.4	113.4 ± 52.0	**<0.001**	178.0 ± 58.0	160.4 ± 59.1	**0.018**	0.728	**0.008**
LA, mm	45.9 ± 5.1	43.8 ± 4.9	**0.001**	47.6 ± 5.8	47.2 ± 5.9	0.480	0.390	**0.039**
RV, mm	25.2 ± 4.4	24.1 ± 3.2	0.093	24.9 ± 4.1	24.1 ± 3.1	0.260	0.877	0.809
LVEDI, mL/m^2^	121.4 [102.6; 144.0]	83.9 [74.0; 108.7]	**<0.001**	124.6 [106.3; 146.7]	103.8 [86.4; 155.2]	**0.019**	0.575	0.051
LVESI, mL/m^2^	89.6 [69.2; 101.8]	50.8 [40.6; 74.5]	**<0.001**	89.0 [69.9; 97.7]	69.2 [56.0; 99.6]	**0.018**	0.854	**0.006**
LAI, mL/m^2^	53.3 [44.6; 66.1]	42.1 [33.9; 54.1]	**<0.001**	58.4 [49.0; 76.6]	53.0 [39.3; 70.2]	**0.023**	0.311	**0.037**
RAI, mL/m^2^	34.7 [29.4; 61.4]	33.8 [28.1; 45.3]	**0.017**	40.9 [29.8; 54.5]	39.7 [27.6; 54.9]	0.712	0.869	0.395
RAV, mL	86.5 ± 39.2	74.3 ± 26.2	**0.013**	86.9 ± 33.4	85.6 ± 38.2	0.593	0.801	0.422
LAV, mL	108.9 ± 34.6	89.0 ± 29.8	**<0.001**	122.9 ± 43.9	112.5 ± 42.1	**0.035**	0.306	**0.040**
IVS, mm	9.9 ± 1.6	10.5 ± 2.1	**0.002**	9.4 ± 1.3	9.8 ± 1.5	0.059	0.434	0.457
LVPW, mm	10.2 ± 1.2	10.4 ± 1.6	0.472	9.8 ± 0.9	9.6 ± 1.2	0.262	0.137	**0.039**
LVEF, %	29.0 [25.0; 31.0]	38.0 [35.0; 45.0]	**<0.001**	32.0 [28.0; 35.0]	32.0 [29.0; 35.0]	0.326	**0.013**	**<0.001**
SV, mL	66.2 ± 16.8	71.1 ± 17.2	0.071	80.1 ± 18.4	73.4 ± 22.7	**0.041**	**0.019**	0.549
MM, g	272.1 ± 59.1	232.5 ± 63.0	**<0.001**	264.6 ± 54.0	246.2 ± 63.7	0.209	0.846	0.411
MMI, g/m^2^	139.4 ± 34.3	119.1 ± 31.3	**<0.001**	134.4 ± 29.1	123.9 ± 29.5	0.124	0.969	0.439
CI, L/min/m^2^	2.1 [1.7; 2.5]	2.4 [1.8; 3.1]	0.072	2.3 [2.1; 3.2]	2.4 [1.7; 3.3]	0.118	0.057	0.969
LVSI	0.69 ± 0.19	0.59 ± 0.07	**<0.001**	0.68 ± 0.08	0.67 ± 0.09	0.834	0.530	**0.008**
E, cm/s	70.7 ± 24.2	53.8 ± 17.4	**0.001**	84.2 ± 35.5	70.0 ± 33.0	**0.014**	0.185	0.131
A, cm/s	61.9 ± 22.2	66.5 ± 19.5	0.291	61.9 ± 23.5	68.1 ± 23.7	0.182	0.824	0.480
E/A	1.40 ± 0.89	0.94 ± 0.63	**0.002**	1.62 ± 0.99	1.27 ± 1.02	0.064	0.505	0.334
e′, cm/s	6.15 ± 1.93	6.21 ± 2.37	0.733	5.65 ± 2.04	6.34 ± 1.96	0.382	0.275	0.636
E/e′	12.2 ± 4.8	9.4 ± 4.7	**0.005**	16.5 ± 8.9	11.0 ± 4.0	**0.012**	**0.046**	0.113
RAA, cm/m^2^	1.79 ± 0.21	1.79 ± 0.21	0.840	1.89 ± 0.26	1.84 ± 0.31	**0.043**	0.231	0.649
RVS, cm/m^2^	1.78 ± 0.19	1.83 ± 0.21	**0.009**	1.86 ± 0.22	1.84 ± 0.26	0.554	0.364	0.915
RVSP, mmHg	31.0 [25.0; 42.0]	29.0 [26.0; 32.0]	**0.035**	37.0 [30.0; 57.0]	31.0 [28.0; 40.0]	0.388	**0.038**	**0.042**

Bold values indicate statistical significance. Values are expressed as mean ± SD and M_e_ [Q_1_; Q_3_]. Abbreviations: A, peak filling velocity during atrial systole; CI, cardiac index; CRT, cardiac resynchronization therapy; E, left ventricle early diastolic filling velocity; e′, early diastolic motion velocity of the mitral annulus; E/A, left ventricle early diastolic filling velocity to peak filling velocity during atrial systole ratio; E/e′, left ventricle early diastolic filling velocity to early diastolic motion velocity of the mitral annulus ratio; GCS, global circular strain; GLS, global longitudinal strain; IVS, interventricular septum; LA, left atrium; LAI, left atrial index; LAV, left atrial volume; LVEDD, left ventricular end-diastolic dimension; LVEDI, left ventricular end-diastolic index; LVEDV, left ventricular end-diastolic volume; LVEF, left ventricle ejection fraction; LVESD, left ventricular end-systolic dimension; LVESI, left ventricular end-systolic index; LVESV, left ventricular end-systolic volume; LVPW, left ventricle posterior wall; LVSI, left ventricular sphericity index; MM, myocardial mass; MMI, myocardial mass index; pts, patients; RAA, ratio of ascending aortic diameter to body surface area; RAI, right atrial index; RAV, right atrial volume; RV, right ventricle; RVFWS, right ventricle free wall strain; RVS, ratio of the Valsalva sinus diameter to the body surface area; RVSP, right ventricle systolic pressure; SR, super-response; SV, stroke volume.

**Table 4:** Basic pre- and post-CRT implantation of gBPS indicators.

Indicators	Pts with CRT SR (n = 39)			Pts Without CRT SR (n = 15)			*p*_1–4_	*p*_2–5_
	Baseline	6 m.	*p*	Baseline	6 m.	*p*		
	1	2	3	4	5	6		
RVID, ms	106.0 [78.0; 133.0]	90.0 [64.0; 112.0]	**0.036**	139.3 [93.0; 182.0]	111.1 [89.0; 146.0]	0.099	**0.040**	0.108
LVID, ms	129.3 [105.0; 155.0]	80.0 [50.0; 123.0]	**<0.001**	134.1 [123.0; 152.0]	113.8 [81.8; 123.0]	0.061	0.779	**0.030**
IVD, ms	93.0 [40.0; 122.0]	55.0 [33.0; 76.0]	**0.003**	45.0 [15.0; 73.0]	37.0 [11.0; 67.0]	0.776	**0.005**	0.167
HBW LV, °	186.0 [126.0; 240.0]	108.0 [72.0; 180.0]	**<0.001**	216.0 [198.0; 253.0]	138.0 [102.0; 180.0]	**0.002**	**0.032**	0.205
Phase standard deviation of the left ventricle walls:								
AW, °	25.0 [16.0; 32.0]	21.0 [9.0; 27.0]	0.055	24.0 [11.0; 34.2]	20.0 [16.0; 23.0]	0.706	0.369	0.794
IW, °	27.0 [15.0; 32.0]	18.0 [11.0; 28.0]	0.214	40.0 [24.0; 44.0]	33.0 [21.0; 44.0]	0.529	**0.021**	0.075
SW, °	45.0 [27.0; 60.0]	26.0 [12.0; 36.0]	**<0.001**	46.0 [39.0; 62.0]	24.0 [17.0; 32.0]	**0.001**	0.816	0.721
LW, °	11.0 [9.0; 15.0]	17.0 [10.0; 24.0]	**0.021**	15.0 [8.0; 22.0]	22.0 [9.0; 44.0]	0.115	0.499	0.523

Bold values indicate statistical significance. Values are expressed as M_e_ [Q_1_; Q_3_]. Abbreviations: AW, anterior wall; CRT, cardiac resynchronization therapy; gBPS, gated blood pool single-photon emission computed tomography; IVD, interventricular dyssynchrony; IW, inferior wall; HBW, histogram bandwidth; LVID, left ventricle intraventricular dyssynchrony; LW, lateral wall; pts, patients; RVID, right ventricle intraventricular dyssynchrony; SR, super-response; SW, septal wall.

## Data Availability

Available upon request.

## Abbreviations

The following abbreviations are used in this manuscript:

6MWDT	Six-minute walk distance test
^99m^Tc-MIBI	^99m^Tc-methoxy-isobutyl-isonitrile
AUC	Area under the ROC curve
BMI	Body mass index
CI	Confidence interval
CRT	Cardiac resynchronization therapy
ECG	Electrocardiography
_e_GFR	Estimated glomerular filtration rate
FC	Functional class
HF	Heart failure
LVEF	Left ventricular ejection fraction
LVPW	Left ventricle posterior wall
M	Mean
M_e_	Median
MI	Myocardial infarction
MPS	Myocardial perfusion scintigraphy
NYHA	New York Heart Association
OR	Odds ratio
SD	Standard deviation
TTE	Transthoracic echocardiography
